# *Mycoplasma pneumoniae* Infection and Progressive Cold Agglutinin Syndrome in a Child: Catastrophic Multiple Cutaneous Necrosis

**DOI:** 10.1002/ccr3.70821

**Published:** 2025-08-26

**Authors:** Bahareh Abtahi‐Naeini, Sharareh Babaei, Sayed Nassereddin Mostafavi, Mahsa Pourmahdi‐Boroujeni, Sara Adibfard

**Affiliations:** ^1^ Pediatric Dermatology Division of Department of Pediatrics, Imam Hossein Children's Hospital Isfahan University of Medical Sciences Isfahan Iran; ^2^ Skin Diseases and Leishmaniasis Research Center Isfahan University of Medical Sciences Isfahan Iran; ^3^ Department of Pediatric Intensive Care Unit, Child Growth and Development Research Center, Research Institute for Primordial Prevention of Non‐Communicable Disease, Imam Hossein Children's Hospital Isfahan University of Medical Sciences Isfahan Iran; ^4^ Department of Pediatric Infectious Diseases, Infectious Diseases and Tropical Medicine Research Center Isfahan University of Medical Sciences Isfahan Iran; ^5^ Student Research Committee Isfahan University of Medical Sciences Isfahan Iran

**Keywords:** child, cold agglutinins, gangrene, *Mycoplasma pneumoniae*, necrosis

## Abstract

Acrocyanosis and gangrene are rare but serious manifestations of CAS caused by MP infection in children. These symptoms may lead to severe complications if not managed appropriately, highlighting the need for clinicians to remain vigilant and provide proper supportive care. In the case we discussed, the administration of FFP, plasmapheresis, corticosteroids, and IVIG led to a satisfactory improvement in the patient's condition.

## 1 Introduction

Cold agglutinins (CAs) are a group of autoantibodies that bind to the antigens on red blood cells (RBCs) at the optimal temperature of 3°C to 4°C It results in the agglutination of erythrocytes and leads to hemolytic anemia [1, 2].

Clinical presentation related to the CAs can be divided into Cold Agglutinin Disease and Cold Agglutinin Syndrome (CAS) [3]. CAS, also known as secondary CA‐mediated hemolytic anemia, is a less common condition that occasionally arises in association with an underlying disease, such as Epstein–Barr virus (EBV) infection, *Mycoplasma pneumoniae* (MP) infection, or aggressive lymphoma [1].

When agglutination occurs due to IgM binding to RBCs and complement activation, the clumped erythrocytes may cause blockage in microcirculation and cause microvascular occlusion syndrome (MVOS), which clinically can lead to multifocal thrombosis and ischemic events in a critical organ, such as the kidney, brain, heart, and eye [4, 5]. Cutaneous manifestations of CAS include a dark, purple to gray discoloration of the skin, Raynaud's phenomenon, acrocyanosis, ischemic gangrene, and cutaneous necrosis [4].

Progressive four‐acral site gangrene is an infrequent manifestation of CAS in the setting of MP infection, especially in children [6].

Here, we report a 9‐year‐old girl with the presentation of acrocyanosis and progressive necrosis of the acral sites after CAS due to MP infection. The importance of the current report lies in the requirement for quick and effective management to prevent these complications.

## 2 Case History/Examination

A 9‐year‐old girl with a previous history of cerebral palsy, seizure disorder, atrial septal defect (ASD), and small patent ductus arteriosus (PDA) was admitted to the hospital due to respiratory distress, a 10‐day fever, and coryza symptoms with a primary diagnosis of pneumonia.

Her fever continued during her hospitalization despite antibiotic therapy with clindamycin and ceftriaxone. Seven days after hospitalization, considering the continuation of the fever and consultation with the infectious department, she was managed as an FUO (fever of unknown origin) case. The workup included a Chest X‐ray, abdominal and pelvic ultrasound, blood and urine culture, Wright and Coombs Wright test, EBV, and Cytomegalovirus (CMV) serology test. Wright and Coombs Wright tests were negative, but IgM serology for Cytomegalovirus and Epstein–Barr virus was positive.

**FIGURE 1:**
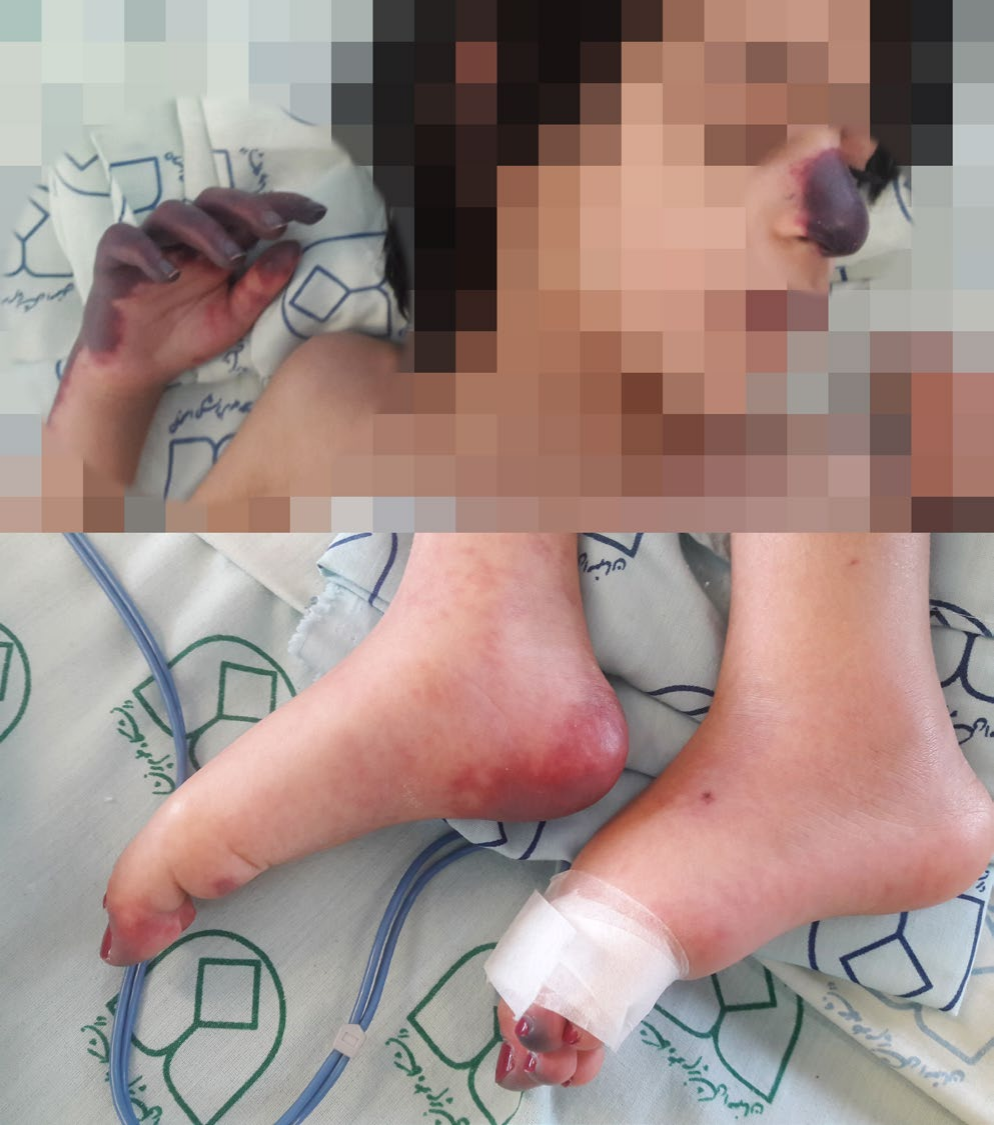
Acrocyanosis and cutaneous necrosis in a 9‐year‐old girl who presented with respiratory distress, fever, and coryza symptoms.

On the 10th day of hospitalization, an episode of a 1‐min tonic–clonic seizure occurred that was self‐limited. On the same day, ecchymotic and purpuric lesions with satellite‐like petechiae appeared at the tip of the nose and then on the extremities (Figure 1). The distal part of the right forearm, right‐hand fingers, left toes, and right heel also had the same lesions. One day later, she developed edema and acrocyanosis in the distal parts of her four extremities. On physical examination, her acral area was cold.

Laboratory investigations showed decreased platelets on the day before the onset of lesions. There was no evidence of hemolysis regarding a normal amount of LDH (lactate dehydrogenase) and hyperbilirubinemia. In addition, in the examination of the peripheral blood smear (PBS), there was no evidence of hemolysis, and no schistocytes were present. Detailed laboratory findings are in Table 1.

**TABLE 1:** Laboratory findings on presentation day.

Test	Result	Unit	Reference interval
*Hematology*			
CBC			
*RBCs*			
RBC	3.48	×10^6^/μL	3.7–5.3
Hemoglobin	13.5	g/dL	10.5–14.0
HCT	30.5	%	33.0–39.0
MCV	87.5	fL	70.0–86.0
MCH	30.7	pg	23.0–31.0
MCHC	35	g/dL	31.0–37.0
*WBCs*			
WBC	8.2	×10^3^/μL	6.0–17.0
*Differential*			
Neutrophil	80	%	20–50
Lymphocyte	14	%	40–65
*Platelets*			
Platelets	75	×10^3^/μL	130–500
ESR (1 h.)	61	mm/h	0–10
*Biochemistery*			
SGOT (AST)	264	U/L	< 38.0
SGPT (ALT)	64	U/L	< 41.0
ALP	266	U/L	36–92
Albumin	2.4	g/dL	3.5–5.5
*Coagulation tests*			
D‐Dimer*	23,880	ng/mL FEU	220–500
FDP*	150	mL/L	< 10
PT	15.5	Sec	11–13.5
INR	1.4	N.A.	0.8–1.1
PTT	43	Sec	25–35
Protein C*	26	IU/dL	45–93
Protein S*	71	IU/dL	41–114
Anti Thrombin I*	90	IU/dL	80–120
Homocysteine*	3.3	μmol/L	3.3–8.3
Fibrinogen*	203	mg/dL	219–516
C_3_*	1.00	g/L	0.88–2.01
C_4_*	0.13	g/L	0.1–0.4
CH50*	100	Hemolytic units	41 to 90
*Electrolytes*			
Sodium	133	mmol/L	135–145
Potassium	4.2	mmol/L	3.7–5.1
Calcium	8	mg/dL	8.8–10.8
*Immunology*			
*Mycoplasma pneumoniae* IgM*	Positive	U/L	Positive > 0.96
ANCA‐C*	12.2	U	Negative: ≤ 19
ANCA‐P*	0.4	U	Negative: ≤ 19
ANA*	0.6	U	Negative: ≤ 1
RA Factor*	Negative		Negative: < 1:80
Anti dsDNA*	8.8	U/mL	Negative: < 10
Anticardiolipin antibody IgM*	55.2	U/mL	Negative: < 12.5
Anticardiolipin antibody IgG*	30.7	U/mL	Negative: < 15
*Inflammatory markers*			
CRP	Positive	mg/L	0–10

*Note:* Items marked with an asterisk (*) indicate significant findings from subsequent days of hospitalization.

Abbreviations: ALP, alkaline phosphatase; ANA, antinuclear antibody; ANCA, antineutrophil cytoplasmic antibodies; Anti‐dsDNA, anti‐double‐stranded DNA; CBC, complete blood count; CH50, 50% hemolytic complement; CRP, C‐reactive protein; ESR, erythrocyte sedimentation rate; FDP, fibrin and fibrinogen‐degradation product; HCT, hematocrit test; INR, international normalized ratio; MCH, mean corpuscular hemoglobin; MCHC, mean corpuscular hemoglobin concentration; MCV, mean corpuscular volume; PT, prothrombin time; PTT, partial thromboplastin time; RA Factor, rheumatoid factor; RBC, red blood cell count; SGOT (AST), aspartate aminotransferase; SGPT (ALT), alanine transaminase; WBC, white blood cell count.

## 3 Methods

The primary clinical diagnosis based on the clinical appearance of the lesion for our patient was cutaneous MVOS. The differential diagnoses for the etiology of this condition were sepsis and disseminated intravascular coagulation (DIC), cryoglobulinemia, cryofibrinogenemia, CAS, autoimmune hypercoagulability states such as antiphospholipid syndrome (APS), etc. So, workups related to differential diagnoses were performed to reach the final diagnosis.

A new laboratory investigation showed an abnormal prothrombotic state. The laboratory panel of coagulation tests was as follows: prothrombin time (PT) = 15.5 s (*N*: 11–13.5), partial thromboplastin time (PTT) = 43 s (N: 25–35), international normalized ratio (INR) = 1.4 (*N*: 0.8–1.1), platelet count (PLT) = 85 × 10^3^/μL (*N*: 150–450 × 10^3^). Also, a marked elevation of erythrocyte sedimentation rate (ESR) (61 mm/h) compared with the first day (18 mm/h) was observed. Cold agglutinin antibody titers were tested, and the result was positive (1:128; *N* < 1:32). A new PBS demonstrated normochromic RBCs with anisopoikilocytosis as target cells, scattered nucleated RBCs and polychromasia, RBC agglutination, neutrophilic predominancy, and decreased platelet count. A respiratory screen panel, including PCR from the nasal and pharyngeal swab, was positive for MP. Therefore, based on clinical examination and laboratory results, the possibility of CAS secondary to MP infection was considered for the child.

Enoxaparin (1 mg/kg/dose) every 12 h, Pentoxifylline (20 mg/kg/day) in 3 divided doses, and Amlodipine (0.1 mg/kg/day) were added to the previous treatment. Despite the added treatment, the lesions at the tip of the nose, right wrist, right heel, and fingers of both hands progressed to gangrene. A few days later, several blisters developed on the right heel and around the gangrene areas of the hands.

**FIGURE 2:**
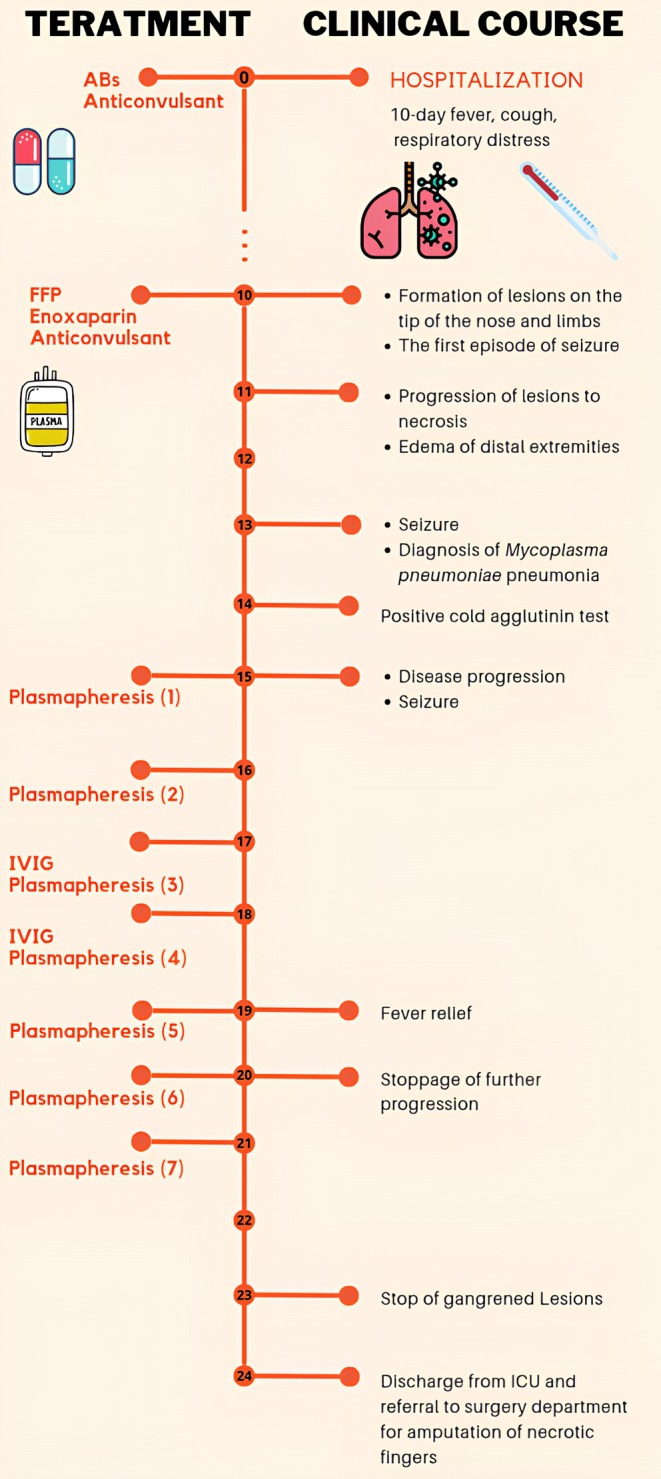
The clinical course and treatments.

Due to the lack of clinical response to the treatment and progression of the lesions to gangrene, heparin at the therapeutic dose, Plasmapheresis, Fresh Frozen Plasma (FFP) (10–15 cc/kg), corticosteroids (1 mg/kg/day), and Intravenous Immunoglobulin (IVIG) (1 mg/kg) were started. The course of the disease and treatment is summarized in Figure 2.

## 4 Conclusion and Results

Five days after the initiation of plasmapheresis, the progression of lesions stopped. The transient ischemia slowly regressed. However, the area of long‐lasting ischemia progressed to a sharply demarcated necrotic lesion. After three months in an outpatient setting, she was referred to the surgery department for the amputation of necrotic fingers.

## 5 Discussion

Here, we described a Persian child with progressive persistence acrocyanosis and peripheral gangrene induced by cold‐agglutinin after MP infection. The clinical manifestations of Respiratory MP vary from asymptomatic to severe complicated pneumonia [7]. MP infection can cause a wide variety of extrapulmonary involvement, such as gastrointestinal, dermatological, neurological, cardiovascular, hematological, musculoskeletal, sensory organ, and urogenital tract manifestations [3, 4, 5].

Dermatological manifestations include nonspecific morbilliform rash, urticaria, Stevens‐Johnson syndrome, Henoch–Schoenlein purpura, etc. Most of the cutaneous manifestations are self‐limited and noncritical [3, 4]. Skin necrosis, progressive acrocyanosis, and gangrene formation are rarely reported manifestations of MP infection, especially in children [6].

Extrapulmonary manifestations may be due to autoimmune reactions or direct invasion of MP [8, 9]. The immune‐related process has been suggested to be responsible for thrombosis [9]. As a complication of MP infection, intravascular coagulation may cause organ damage, such as skin, brain, lung, and kidney infarction [10].

CA autoantibodies, which are usually IgM subtypes, bind to the antigens of erythrocytes and lead them to agglutinate after exposure to cold temperatures [2]. These clumped RBCs may cause blockage in microcirculation, leading to MVOS [4, 11]. There are limited reports of cutaneous manifestations in the setting of MP pneumonia, and among them, a few are caused by CAS (Table 2).

**TABLE 2:** Reports of acral cutaneous manifestations in the setting of *Mycoplasma pneumoniae* infection.

Authors/year/ref	Age (year)/gender	Clinical manifestation	Paraclinical manifestation	Laboratory data	Treatment	Outcome
Inga Marie Nilsson Alf Rausing, 1971 [10]	3/F	Fever Vomiting Respiratory distress Tachycardia Somnolence Prostration Generalized convulsions Oliguria and then anuria Rectal bleeding *Skin*: Central and peripheral cyanosis Petechiae in the distal parts of the legs Gangrene of the legs	*Chest X‐ray*: Right‐sided pneumonia Atelectasis Pneumothorax	*During hospitalization, the culture of bronchial secretion and blood*: + for Candida *Mycoplasma pneumoniae antibodies*: +	Antibiotics Antifungals Hemodialysis Heparin Fresh blood ACTH Exchange transfusions	Expired
Bernadett Mosdósi Zoltán Nyul, 2017 [4]	9/F	Previous history of asthma Unproductive cough Subfebrile Crepitation above the middle lobe of the right lung *Skin*: Acrocyanosis Painful, bluish discoloration of the fingers (Worsening in cold temperatures) Small cutaneous necrosis of the digital phalanx of the fourth finger	*Chest X‐ray*: Pneumonia in the right lobe *Doppler examination*: NL *Capillary microscopy*: Ramified capillaries *Laser Doppler Flowmetry (LDF) and Laser Speckle Contrast Analysis (LASCA)*: Severely reduced basal blood flow on the affected fingers with delayed vasodilatation after local heating	*Cryoglobulins*: − *IgM (auto‐agglutinines) and C3d*: + *Anti‐nucleosome antibody*: ↑ *Mycoplasma IgM and IgA antibodies*: + *Mycoplasma IgG antibody*: +	Antibiotics Fluticasone propionate Salbutamol inhalationPentoxifyllinee	Healed/without any complications
Weizhen Guo Iris Wai Sum Li, 2017 [12]	1/F	Fever Non‐productive cough Pharyngitis/tonsilitis *Skin*: Redness and swelling over the middle phalanx of the right index finger and hand (Swelling progressed to abscess with pus discharge)	*Chest X‐ray*: Bronchopneumonia *X‐ray*: Soft tissue swelling of the index finger without bone erosion	*Mycoplasma pneumoniae IgM antibodies*: + *Abscess Pus Culture*: + for *Chromobacterium violaceum* *HIV antibodies and neutrophil dysfunction*: −	Antibiotics Surgical drainage of abscess	Healed/without any complications
Jung Hee Woo Jung Hyun Kwon, 2018 [9]	8/M	Cough Fever Respiratory distress Neck and chest pain *↑Capillary refill time* *↓ Sensation in the left index finger* *Skin*: Discoloration at the tip of his left index finger	*Chest X‐ray*: Lingular consolidation in the right upper lobe Pneumonia Atelectasis Pneumothorax *Doppler ultrasonography*: NL *Angiography of the left upper extremity*: Thrombotic obstruction of the left radial artery	*Mycoplasma pneumoniae IgM*: + *Anti‐Phospholipid antibodies*: + *Cold agglutinin*: +	Antibiotics Steroids Low‐molecular‐weight heparin (LMWH) Systemic urokinase Thrombectomy Nitroglycerin Heparin Aspirin Clopidogrel	Healed/without any complications
Patrick M. Meyer Sauteur, MD Martin Theiler, MD, 2019 [8]	11/F	Low‐grade fever Bilateral crackles of the lungs *Skin*: Erythematous macules in the periungual and pulp areas of all fingers	*Chest X‐ray*: Interstitial lung disease	*Cryoglobulins*: − *Mycoplasma pneumoniae IgM and IgG*: +	Acetaminophen Antibiotics	Healed/without any complications
Devon W. Hahn Claire E. Atkinson, 2021 [13]	10/M	Fever Sore throat Non‐productive cough Respiratory distress Neck and back pain Severe sepsis and pneumonia Endocarditis *Skin*: Edema in the distal extremities Diffuse non‐tender scalp edema	*Chest X‐ray and CT*: Left upper lobe consolidation Bilateral pulmonary emboli Multiple splenic infarcts Non‐occlusive venous thrombus in the internal jugular tributary vein *Transesophageal echocardiogram*: A small echogenic, slightly mobile mass along the right side of the ventricular septum *Doppler ultrasound of the extremities*: Multiple deep venous thromboses in bilateral lower and right upper extremities	*Blood culture*: One of two was + for *Rothia dentocariosa* *Immunoglobulin ↑* *Presence of red blood cell agglutination* *Lupus ANA with nuclear speckled and cytoplasmic patterns*: + *Mycoplasma pneumoniae IgM and IgG*: +	Antibiotics Vitamin K Heparinization Low‐molecular‐weight heparin (LMWH) Enoxaparin	Healed/without any complications
Murugan Sudhakar Vichithra Mohandoss, 2021 [6]	8/M	Fever Pulslessness in both lower limbs *Skin*: Maculopapular rash over the trunk Blackish discoloration of the right foot Dry gangrene in the distal third of the right foot	*Chest X‐ray*: Homogeneous opacity in the left middle and lower zones *CT angiography of the abdomen and lower limbs*: Infarcts in both kidneys and multifocal thrombotic occlusion in bilateral popliteal arteries	*Autoagglutination of red blood cells at room temperature*: + *Anti‐C3d*: 2 + *Cold agglutinin*: + *Mycoplasma pneumoniae IgM*: + *EBV and CMV IgM*: + *ANA test*: +	Antibiotics Low molecular weight heparin (LMWH) Aspirin Amlodipine Unfractionated heparin Methylprednisolone Prednisolone Prostaglandin E1	Surgical amputation of the right midfoot
Our case	9/F	Fever Productive cough Respiratory distress Soft systolic murmur (2/6) Seizure *Skin*: Ecchymotic lesions on the tip of the nose and distal extremities with acral retiform purpura Gangrene lesions Popular rashes on the lower limbs and around the eyes	*Chest X‐ray*: Patchy diffuse lung opacity Pleural effusion Collapse *Echocardiography*: Dilated RV and RA, PDA, large ASD, mild Pulmonary Hypertension due to heart failure, and microscopic polyangiitis *Abdominopelvic ultrasound*: NL *Brain‐CT without contrast*: NL *Doppler ultrasound of iliac and femoral vessels*: NL	*Blood culture*: + for Viridans Streptococci *Cold agglutinin*: + *Mycoplasma pneumoniae IgM*: + *EBV and CMV IgM*: + *ANA test*: −	Acetaminophen Methylprednisolone Ventolin Antivirals Antibiotics Anticonvulsants Enoxaparin FFP Pack cell Platelet Plasmapheresis IVIG Pentoxifylline Amlodipine	Amputation of necrotic fingers

Abbreviations: ANA, antinuclear antibody; CMV, cytomegalovirus; EBV, Epstein–Barr virus; F, female; FFP, fresh frozen plasma; IVIG, intravenous immunoglobulin; M, male; NL, normal.

Although some cases had a similar condition to ours, they did not show gangrene and necrosis in their extremities. In one of these cases, MP infection ended in swelling, redness, and abscess [12]. Another one presented discoloration of fingers [9]. Also, Meyer Sauteur et al. reported an 11‐year‐old female with red fingers and erythematous macules on her fingers [8]. Additionally, an 8‐year‐old male was described as having discoloration of his finger due to thrombosis after MP infection; another one reports a 10‐year‐old male with edema in distal extremities and scalp with the same cause as the previous case [9, 13].

In some other studies, similarly to our case, patients demonstrated symptoms of gangrene and necrosis in the extremities. Sudhakar et al. reported an Indian 8‐year‐old boy who had a maculopapular rash, acral discoloration, and finally gangrene of his foot due to CAS secondary to MP pneumonia [6]. In another case, Nillson et al. described a 3‐year‐old girl who expired in the clinical setting of intravascular coagulation due to MP infection. Dermatological manifestations in this child included peripheral cyanosis, petechiae, and foot gangrene [10]. In a similar case, Mosdósi et al. reported a 9‐year‐old female with acrocyanosis and necrosis of her fingers, which was caused by MP pneumonia [4].

In a few cases, gangrene and necrosis in their extremities healed without complications, but in some of them, including our case, surgical amputation was performed [4, 6, 10].

## Author Contributions

**Bahareh Abtahi‐Naeini:** data curation, writing – review and editing. **Sharareh Babaei:** data curation, writing – review and editing. **Sayed Nassereddin Mostafavi:** data curation. **Mahsa Pourmahdi‐Boroujeni:** writing – original draft, writing – review and editing. **Sara Adibfard:** writing – original draft, writing – review and editing.

## Ethics Statement

The Ethics Committee of the Isfahan University of Medical Sciences in Isfahan, Iran, approved this report's ethical content (IR.ARI.MUI.REC.1401.248).

## Consent

Written informed consent was obtained from the patient's parents for the publication of clinical information and photographic material related to this case. The parents were informed that the case details would be published on an open‐access basis, allowing unrestricted access worldwide. They acknowledged their understanding that once published, the information may be freely available and used for educational and research purposes.

## Conflicts of Interest

The authors declare no conflicts of interest.

## Data Availability

Upon written request, the corresponding author will provide the data used to support the findings and conclusions.
